# Comparision of Efficacy and Safety of Orlistat vs Placebo in Obese Patients in Pakistan

**DOI:** 10.7759/cureus.9775

**Published:** 2020-08-16

**Authors:** Asghar Hussain Syed, Areeba Meraj, Laxman Bhandari, Faria Khan, Anam Shaikh, Kanza Baig, Besham Kumar

**Affiliations:** 1 Family Medicine, Ziauddin University, Karachi, PAK; 2 Internal Medicine, Civil Hospital Karachi, Karachi, PAK; 3 Internal Medicine, Nidan Hospital, Lalitpur, NPL; 4 Internal Medicine, Dow University of Health Sciences, Karachi, PAK; 5 Internal Medicine, Jinnah Sindh Medical University, Karachi, PAK; 6 Internal Medicine, Jinnah Postgraduate Medical Centre, Karachi, PAK

**Keywords:** obesity, orlistat, pakistan

## Abstract

Introduction

Obesity has become a rising health concern in Pakistan. Several primary measures, such as dietary restrictions and regular exercise, are taken to limit the severe consequences of obesity. Still, such measures alone have been proven to be often short term and therefore, inadequate. In this study, we will evaluate the role of pharmacological management via orlistat in obesity.

Methods and Materials

A total of 120 patients were enrolled in this study and were divided randomly into two groups (drug arm and placebo arm) of 60 patients each. A standard dose of 120 mg capsules of orlistat given three times a day was administered to the participants of the drug arm group. A placebo was given to the participants in the second group. Baseline investigations were conducted on day 0 of the study, and follow-up visits were planned to take place in week 24 for all participants. Any side effects or adverse events were inquired about and documented in these visits.

Results

In the orlistat arm, we noted a significant reduction in both body mass index (BMI) (0.04) and waist circumference (0.04). Reduction in weight was more in the orlistat arm than in the placebo arm. However, it is non-significant when compared between day 0 and week 24. Adverse events, such as oily spotting (31.91%), flatus with discharge (27.65%), faecal urgency (25.53%) and fatty stool (35.553%), were significantly higher in the orlistat arm.

Conclusion

Orlistat has shown positive results in reducing weight, BMI and waist circumference; however, patients should be counselled about potential side effects caused by the mechanism of action of Orlistat.

## Introduction

Obesity has become a rising health concern in Pakistan, where the prevalence of obese people rose from just 28% in 2011 to an alarming 37.8% within seven years, according to the 2018 National Nutritional Survey (NNS) [1]. This is particularly concerning because obesity has been a leading contributor to increased morbidity as well as mortality. Obesity has been associated with comorbidities, such as hypertension, type II diabetes mellitus, dyslipidaemia and endocrinal abnormalities [2]. It is also reportedly associated with higher mortality from some types of malignancies of the colon, oesophagus, rectum and breast.

Several primary measures, such as dietary restrictions and exercise, are taken to limit the severe consequences of obesity. Still, such measures alone have been proven to be often short term and therefore, inadequate [3]. Thus, there is a need for pharmacotherapy adjunct to lifestyle modifications in these patients [3,4]. Previously used anti-obesity drugs acted on the central nervous system to suppress appetite and reduce food intake, but these drugs, amphetamine, for example, produced significant adverse effects [5]. The association of fenfluramine with cardiovascular side effects is another example and led to their withdrawal from weight loss therapy. 

Orlistat is a newer drug that has been reportedly effective in reducing weight, with minimal adverse effects [6]. Orlistat is a lipase inhibitor that acts on the intestines to reduce fat absorption, which helps in limiting weight gain. Despite the rising prevalence of obesity in Pakistan, there is an unfortunate lack of studies on the efficacy and safety of various pharmacological options available today. This study aims to evaluate the effectiveness and safety of orlistat in obese patients in Pakistan.

## Materials and methods

After obtaining approval from the Institutional Ethics Committee in Pakistan, this study was carried out at the Outpatient Department of an Endocrinology Hospital. This was a prospective, randomised, single-blind, parallel-group study that was carried out over a period of 24 weeks.

The inclusion criteria included both male and female patients, between 18 and 60 years of age having a body mass index (BMI) > 30 kg/m^2^. The exclusion criteria comprised patients with previously known obesity due to endocrinological reason, patients with clinically significant cardiovascular, respiratory or hepatobiliary disorder, patients with chronic diarrhoea and patients using other medications that can potentially alter body weight and/or lipid levels. Pregnant and lactating women were also excluded from the study.

A total of 120 patients were enrolled in the study after their informed consent, and were randomised into two groups of 60 patients each. A standard dose of 120 mg capsules of orlistat given three times a day was selected for the drug arm. Patients in group I received this standard dose, one hour each before each meal. Patients in group II received a placebo three times, also one hour before each meal. Irrespective of the group allotted, every patient was counselled about the importance of lifestyle modifications, including healthy dietary habits and exercise, in weight reduction and maintenance.

Before the administration of the drug, a baseline clinical examination of all participants, including weight and BMI, and laboratory investigations like lipid profile and blood sugar level were carried out and duly recorded. Follow-up visits were planned to take place in week 24, respectively, and any side effects or adverse events were inquired about and documented in the follow-up visits. Mean was compared for day 0 and week 24 for both orlistat and placebo arm, using the t-test. Adverse events were compared using chi-square test. A P value less than 0.05 indicates that the difference between study and control group is significant enough to discard the null hypothesis. 

## Results

A total of 120 patients were enrolled in the study; however, only 87 participants completed the study and were included in the analysis. A total of 42 (48.27%) participants were male and 45 (51.73%) participants were female. The mean age of the participants was 36.19 ± 4.01 years.

In the orlistat arm, 47 participants completed the study, while 11 participants were lost to follow-up, and 2 participants withdrew from the study because of an adverse event. In the 47 participants who completed the study, there was a significant reduction in BMI (0.04) and waist circumference (0.04). However, weight reduction was non-significant (0.10). There was also a significant reduction in total cholesterol (0.0001) and low-density lipoprotein (0.01) (Table 1).

**Table 1:** Parameters in Orlistat Arm BMI, body mass index; LDL, low-density lipoprotein; HDL, high-density lipoprotein; SD, standard deviation

Parameter for orlistat arm (n = 47)	Day 0 (mean ± SD)	Week 24 (mean ± SD)	P value
Weight (kg)	93.16 ± 14.15	88.72 ± 12.11	0.10
BMI (kg/m^2^)	34.18 ± 4.52	32.51 ± 3.51	0.04
Waist circumference (cm)	95.02 ± 10.98	91.22 ± 6.63	0.04
Total cholesterol (mg/dl)	225.19 ± 14.72	211.20 ± 10.14	0.0001
Total triglyceride(mg/dl)	164.02 ± 12.51	162.91 ± 10.18	0.63
LDL (mg/dl)	135.21 ± 10.01	130.16 ± 9.13	0.01
HDL (mg/dl)	36.19 ± 4.99	35.79 ± 5.02	0.69
Random blood glucose level (mg/dl)	112.20 ± 10.03	109.27 ± 7.85	0.11

In the placebo arm, 40 participants completed the study, while 20 participants were lost to follow-up. The difference in parameter between day 0 and week 24 in the placebo group was non-significant (Table 2).

**Table 2:** Parameters for Placebo Arm BMI, body mass index; LDL, low-density lipoprotein; HDL, high-density lipoprotein; SD, standard deviation

Parameter for placebo arm (n = 40)	Day 0 (mean ± SD)	Week 24 (mean ± SD)	P value
Weight (kg)	94.54 ± 15.75	92.04 ± 13.39	0.44
BMI (kg/m^2^)	34.21 ± 5.29	33.61 ± 4.12	0.56
Waist circumference (cm)	96.11 ± 12.12	94.51 ± 9.98	0.52
Total cholesterol (mg/dl)	221.51 ± 15.61	217.12 ± 13.51	0.18
Total triglyceride (mg/dl)	165.21 ± 12.71	161.82 ± 13.18	0.24
LDL (mg/dl)	135.21 ± 11.07	133.00 ± 14.14	0.43
HDL (mg/dl)	37.22 ± 2.52	36.58 ± 2.99	0.03
Random blood glucose level (mg/dl)	113.98 ± 9.62	112.13 ± 8.15	0.35

The mean reduction of weight, BMI and waist circumference between day 0 and week 24 were more in the orlistat arm compared to the placebo arm (Table 3).

**Table 3:** Mean Change in Parameters between Day 0 and Week 24 BMI, body mass index; LDL, low-density lipoprotein; HDL, high-density lipoprotein

Mean change in parameters between day 0 and week 24	Orlistat arm	Placebo arm
Weight (kg)	-4.44	-2.5
BMI (kg/m^2^)	-1.67	-0.6
Waist circumference (cm)	-3.80	-1.6
Total cholesterol (mg/dl)	-14.19	-4.39
Total triglyceride (mg/dl)	-1.11	-3.39
LDL (mg/dl)	-5.05	-2.21
HDL (mg/dl)	+0.4	+0.64
Random blood glucose level (mg/dl)	-2.93	-1.85

Adverse events, such as oily spotting, flatus, faecal urgency and fatty stool, were significantly higher in the orlistat arm. Diarrhoea and faecal incontinence were numerically higher but not statistically significant (Table 4).

**Table 4:** Reported Adverse Events

Adverse event	Orlistat arm (n = 47)	Placebo arm (n = 40)	P value
Oily spotting	15 (31.91%)	1 (2.5%)	0.0001
Flatus with discharge	13 (27.65%)	2 (5%)	0.005
Faecal urgency	12 (25.53%)	1 (2.5%)	0.006
Fatty stool	12 (25.53%)	0	0.001
Diarrhoea	6 (12.76%)	1 (2.5%)	0.07
Faecal incontinence	3 (6.38%)	1 (2.5%)	0.7

## Discussion

A sedentary lifestyle, in addition to unhealthy eating habits, is contributing to the increased prevalence of obesity worldwide. Obesity is a known causative factor for increased mortality and morbidity. It has been linked to multiple diseases, such as cardiovascular ailments, altered blood lipid, increased blood pressure, stroke, diabetes mellitus and various forms of cancer [2]. Therefore, it is essential to manage and reduce weight. Different management techniques are available, such as lifestyle changes (healthy diet, regular exercise), behavioural therapy, pharmacotherapy and surgery. Pharmaceutical intervention is needed in patients where lifestyle interventions have either failed or are not possible [2]. Medications for obesity are broadly divided into two categories: drugs for short-term use and long-term use. FDA-approved medications falling into the first category include diethylpropion, phendimetrazine, benzphetamine and phentermine. Drugs for long-term use include orlistat, phentermine/topiramate extended-release (ER), lorcaserin, naltrexone/bupropion ER and liraglutide [7]. Out of all these drugs, orlistat is the only FDA-approved medication available in Pakistan.

In our study, mean weight reduction was 4.44 kg, which is comparable to other previous studies. Bakris et al. in his study showed a weight loss of 5.4 kg [8]. Broom et al. showed a mean weight loss of 5.8 kg [9]. A study from India, by Jain et al., showed a mean weight loss of 4.65 kg [10]. Mean BMI reduction was also comparable between our research and other literature available. Our study showed a mean BMI reduction of 1.67 kg/m^2^; meanwhile, Bakris et al. showed a mean decrease of 1.9 kg/m^2^ and Jain et al. showed 1.91 kg/m^2 ^[8,10].

The mean total cholesterol in the orlistat arm at day 0 was 225.19 ± 14.72 mg/dl, which was reduced to 211.20 ± 10.14 mg/dl at week 24. The mean reduction was 14.19 mg/dl. Jain et al. showed a mean reduction of 10.68 mg/dl [10]. Other studies showed a mean decrease of 12 mg/dl and 11.6 mg/dl [5,6]. Low-density lipoprotein reduction in our study was 5.5 mg/dl. Finer et al. showed a similar decrease of 4.2 mg/dl, while Jain et al. showed a reduction of 5.87 mg/dl [5,10]. A meta-analysis of 33 randomised control trials showed that total- and low-density lipoprotein cholesterol-lowering were inversely related to the duration of orlistat treatment and positively with body weight change [11]. Orlistat was well tolerated in our study, and side effects were mostly associated with the gastrointestinal system, which can be attributed to its mechanism of action.

Keeping in view the burden of obesity in our country, pharmacotherapy must be considered in suitable patients for reducing the load of morbidity and mortality related to obesity. Reducing obesity may provide not only health benefits but economic benefits as well.

## Conclusions

From this study, it is concluded that orlistat is an effective and well-tolerated option for weight reduction when combined with appropriate lifestyle modifications. It not only reduces weight, BMI, and waist circumference, but it has also proven to have a beneficial impact on blood lipid levels. Thus, the beneficial effects of orlistat in our population are promising, and they should be studied further to help us better understand its role in reducing obesity and comorbidities, which will, consequently, help reduce the burden on the healthcare system.

## References

[1] Unicef Unicef (2020). UNICEF. National Nutrition Survey 2018 - key findings report. https://www.unicef.org/pakistan/reports/national-nutrition-survey-2018-key-findings-report#:~:text=The%202018%20Pakistan%20National%20Nutrition,environmental%2C%20anthropometric%20and%20biochemical%20indicators.

[2] Spiegelman BM, Flier JS (2001). Obesity and the regulation of energy balance. Cell.

[3] Christensen R, Kristensen PK, Bartels EM, Bliddal H, Astrup A (2007). Efficacy and safety of weight- loss drug rimonabant: a meta-analysis of randomised trials. Lancet.

[4] Padwal RS, Majumdar SR (2007). Drug treatments for obesity: orlistat, sibutramine, and rimonabant. Lancet.

[5] Finer N, James WP, Kopelman PG, Lean ME, Williams G (2000). One year treatment of obesity: a randomised, double blind, placebo-controlled, multicentre study of orlistat, a gastrointestinal lipase inhibitor. Int J Obes Relat Metab Disord.

[6] Davidson MH, Hauptman J, DiGirolamo M (1999). Weight control and risk factor reduction in obese subjects treated for 2 years with Orlistat: A randomised controlled trial. JAMA.

[7] Velazquez A, Apovian CM (2018). Pharmacological management of obesity. Minerva Endocrinol.

[8] Bakris G, Calhoun D, Egan B, Hellmann C, Dolker M, Kingma I; orlistat and resistant hypertension investigators (2002). Orlistat improves blood pressure control in obese subjects with treated but inadequately controlled hypertension. J Hypertens.

[9] Broom I, Wilding J, Stott P, Myers N, UK Multimorbidity Study Group (2002). Randomised trial of the effect of orlistat on body weight and cardiovascular disease risk profile in obese patients: UK Multimorbidity Study. Int J Clin Pract.

[10] Jain SS, Ramanand SJ, Ramanand JB, Akat PB, Patwardhan MH, Joshi SR (2011). Evaluation of efficacy and safety of orlistat in obese patients. Indian J Endocrinol Metab.

[11] Sahebkar A, Simental-Mendía LE, Reiner Ž, Kovanen PT, Simental-Mendía M, Bianconi V, Pirro M (2017). Effect of orlistat on plasma lipids and body weight: a systematic review and meta-analysis of 33 randomised controlled trials. Pharmacol Res.

